# Microvolt T-wave alternation in left ventricular hypertrophy patients

**DOI:** 10.1186/1753-6561-7-S1-P8

**Published:** 2013-01-30

**Authors:** Goar Arutyunyan, DA Tsaregorodtsev, IR Bukiya

**Affiliations:** 1 I.M. Sechenov First Moscow Medical State University, Moscow, Russia

## Background

Sudden cardiac death (SCD) is responsible for 11% of all deaths and 50% of all cardiovascular deaths. Hypertrophic cardiomyopathy (HCM) is the most common cause of SCD among the young patients. SCD is often the first manifestation of the disease. There are 5 major risk factors for SCD in patients with HCM. However, no single risk factor can be used as a screening test in assessment of SCD. Nowadays new non-invasive risk factors, such as T-wave alternation (TWA), are being studied. They may provide important information on the patient’s susceptibility to life threatening arrhythmias.

## Methods

The study involved 33 hypertensive heart disease (HHD) patients and 22 HCM patients. All patients underwent echocardiography test (ECHO) and 24h Holter monitoring with mTWA evaluation using the modified moving average method. The evaluation involved maximum mTWA figures in 24h (mTWA max), mTWA figures at the heart rate of 100/min (mTWA 100) and at 5:00 am (mTWA 5:00).

## Results

The common group demonstrated a weak direct correlation between the thickness of ventricular septum (IVSth) and mTWA max, as well as a weak reverse correlation between the left ventricular posterior wall thickness (LVPWth) and mTWA 100. These correlation were revealed primarily in HHD patients who demonstrated correlation between mTWA max and IVSth, between mTWA 5:00 and IVSth, LVPWth, as well as between mTWA 100 and LVPWth. The HCM patients did not demonstrate correlation between mTWA and the thickness of left ventricular wall. The patients with non-obstructive HCM (n=15) demonstrated significantly higher mTWA 100 in comparison to the patients with obstructive HCM (n=5). The presence of higher SCD risk factors in the HCM patients (n=6) did not influenced mTWA figures. The mTWA figures in the HHD and HCM patients differed insignificantly, with the only exception of higher mTWA 5:00 in the HHD in comparison to the HCM group.

## Conclusions

mTWA figures correlate with the degree of left ventricular hypertrophy, however the revealed correlations are weak. The causes of lower MTWA 100 in patients with more intensive hypertrophy (reverse correlation) as well as in patients with obstructive HCM in comparison with non-obstructive HCM call for additional studies.

